# Influence of psychosocial factors and parent–student relationships on the academic engagement of TCM students: a structural equation modeling and multi-criteria decision-making framework

**DOI:** 10.3389/fpsyg.2025.1619509

**Published:** 2025-07-22

**Authors:** Liang Shaoshuai, Peng Qian, Zhang Yiwen, Xu Meiru

**Affiliations:** Shandong College of Traditional Chinese Medicine, Yantai, Shandong, China

**Keywords:** emotional quotient, adversity quotient, psychological resilience, parent–student relations, academic engagement, VIKOR-AHP, MCDM

## Abstract

**Background:**

Students with high IQs often underperform in practice-oriented fields like Traditional Chinese Medicine (TCM) because they lack the emotional regulation, stress management, and interpersonal skills needed to realize their academic potential. Recent research underscores the critical role of psychosocial competencies—emotional quotient (EQ), adversity quotient (AQ), and psychological resilience (PR)—in bridging cognitive potential and real-world academic success. Furthermore, the interplay between EQ, AQ, PR, and parent-student relationships, an underexplored dimension in higher education, may serve as a stabilizing force against academic stressors.

**Methods:**

The study involved 428 Traditional Chinese Medicine (TCM) students who underwent comprehensive evaluations to assess EQ, AQ, PR, parent-student relationships, and academic engagement. Structural equation modeling (SEM) via SmartPLS analyzed causal pathways, while the VIKOR-AHP method addressed MCDM by (1) weighting criteria via AHP and (2) ranking interventions via VIKOR’s compromise-ranking, ensuring practical solutions amid real-world constraints.

**Results:**

This study examined the impact of EQ, AQ, PR, and parent-student relationships on the academic engagement of TCM students. The results indicated that engagement was directly influenced by EQ, while it was indirectly enhanced through improved PR and AQ. During academic challenges, parental-student relationships emerged as a significant moderator. EQ and AQ were identified as the most critical factors in promoting academic engagement. To ensure robustness, a sensitivity analysis was performed to assess how variations in experts’ weights influenced the outcomes, thereby validating the stability and reliability of the results under uncertainty.

**Implications:**

This study, grounded in Csikszentmihalyi’s flow theory, proposes specific strategies to enhance academic engagement. While students often possess adequate cognitive abilities, they require improved emotional regulation and stress management skills to convert potential into achievement. Educational institutions should integrate emotional quotient training with traditional cognitive curricula, alongside resilience-building programs and efforts to foster parent-student collaboration. These non-cognitive approaches not only improve academic performance but also promote personal adaptability. The findings advocate for comprehensive educational frameworks that combine cognitive and psychosocial development to optimize student engagement.

## 1 Introduction

Students with high emotional quotient (EQ) yet poor academic performance, commonly referred to as “underachievers,” provide a significant educational issue. These students exhibit considerable cognitive potential yet do not convert it into academic achievement. Multiple variables contribute to this phenomenon, encompassing personal, educational, and environmental effects (Ononye et al., 2022). Emotional quotient and psychological resilience are essential in closing this gap, as they profoundly affect academic performance and personal growth (Merzaq et al., 2024). Comprehending these elements is essential for formulating appropriate strategies to assist these students.

Emotional quotient (EQ) is a key predictor of academic success. It improves students’ emotional regulation, resulting in enhanced stress management and interpersonal interactions, both of which are crucial for academic success (Merzaq et al., 2024). Research shows a strong positive correlation between higher EQ, greater psychological resilience (the ability to adapt to adversity), and academic performance. Despite their cognitive advantages, gifted students remain vulnerable to academic underperformance when deficient in noncognitive skills (e.g., emotional quotient, psychological resilience), as highlighted by Larionova et al. (2021). This study highlights the mediating role of adversity quotient (AQ) and psychological resilience in the academic engagement of Traditional Chinese Medicine (TCM) students, proposing a novel conceptual framework.

Multiple factors may affect the correlation between emotional quotient (EQ) and academic engagement, particularly through parent-student relationships as a moderating variable. Parenting styles, parental involvement, and students’ psychological needs collectively shape academic engagement. Supportive parental involvement positively impacts students’ academic engagement by providing emotional and educational support that enhances motivation and psychological resilience (Anierobi et al., 2024). For high-ability students in particular, parental expectations and involvement may moderate the link between emotional quotient (EQ) and academic engagement (Phillipson, 2010). However, this relationship varies significantly depending on individual student needs and contextual factors. Phillipson’s (2010) research suggests that low-achieving students require tailored parental support to improve outcomes. This study examined the moderating role of parent-student relationships in the association between emotional quotient (EQ) and academic engagement.

Traditional Chinese Medicine (TCM) students face unique emotional and psychological challenges due to cultural expectations and a rigorous curriculum (Lu et al., 2024). These factors contribute to heightened stress levels, impacting their mental well-being and academic performance (Zhang et al., 2024). Understanding these challenges is crucial for developing effective support systems. Psychological resilience capabilities—such as perseverance, self-confidence, and ambition—are vital for success in TCM. These capabilities, alongside a supportive family environment, enhance students’ overall quality and readiness for the profession (Yang et al., 2023). As job market challenges grow, TCM students must develop skills for career exploration and adaptability, emphasizing the importance of noncognitive capabilities in their training (Li et al., 2023). TCM education emphasizes not only theoretical knowledge but also noncognitive competencies essential for patient care. Masten et al. (2021) define psychological resilience as a developmental capacity shaped by individual and contextual assets, aligning with our findings on AQ and parental influence in TCM education. This holistic approach is crucial for developing adaptable practitioners capable of navigating clinical uncertainties. The curriculum and future career path for Traditional Chinese Medicine (TCM) students are significantly more demanding than those in other academic disciplines.

The Weighted Linear Combination approach for ranking innovation initiatives significantly influences project rankings (Mizla et al., 2021). Silva et al. (2018) illustrate that the SMARTS-Choquet methodology correlates innovation metrics with sustainability aspects, so showing how MCDM can guide investment choices based on extensive criteria. The integration of Multi-Criteria Decision Making (MCDM) methodologies, notably the Analytic Hierarchy Process (AHP) and SmartPLS, has facilitated innovative decision-making approaches across various disciplines. The research incorporated the advantages of both linear and nonlinear relationship analysis techniques.

This study is notable as it represents the inaugural examination to thoroughly examine the factors affecting academic engagement among students majoring in Traditional Chinese Medicine. The study’s development of an expanded academic engagement model will enhance comprehension of the emergence of students’ mental health issues from the viewpoints of both experts and institutions, facilitating the investigation of the factors that influence the improvement of students’ academic performance. The findings will assist policymakers, educators, and administrators in improving TCM students’ academic engagement.

## 2 Literature review

**Figure 1:**
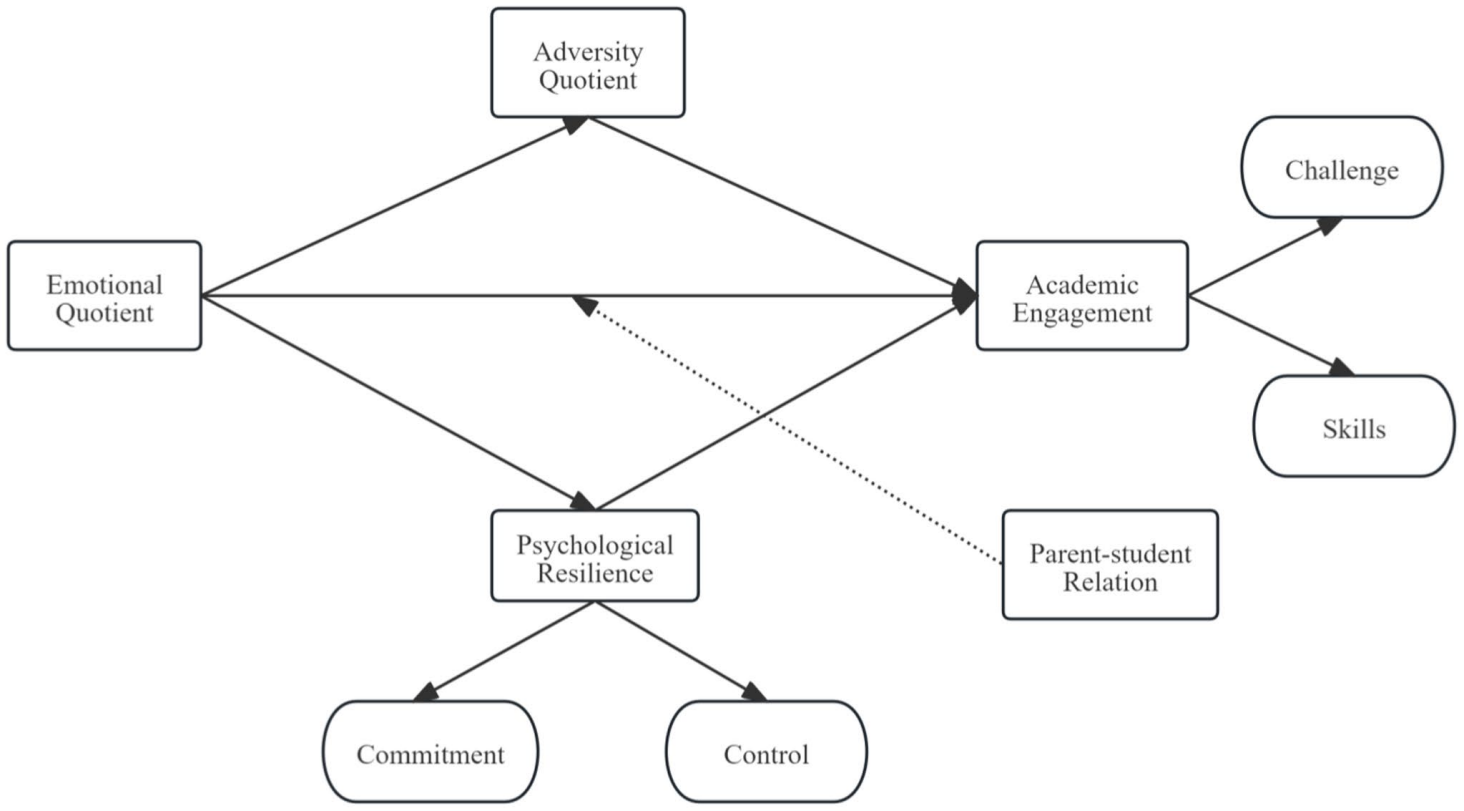
Conceptual framework.

The conceptual framework was established based on Csikszentmihalyi’s flow theory, which asserts that an optimal experience, or “flow,” occurs when there is a balance between the demands of an activity and an individual’s skills (Schmidt et al., 2014). This equilibrium is essential for attaining a condition of profound involvement and pleasure. Flow Theory underscores how optimal learning environments enhance TCM students’ engagement by fostering deep absorption and enjoyment in academic tasks. Such flow experiences strengthen motivation, concentration, and well-being—key noncognitive factors for effective learning. Balancing challenge and skill while providing support cultivates sustained academic involvement and professional growth (Shernoff and Twersky, 2022). The following Figure 1 showed the conceptual framework.

### 2.1 Emotional quotient and academic engagement

The correlation between emotional quotient (EQ) and academic engagement of students majoring in TCM is a complex subject that encompasses several psychological variables, such as adversity quotient (AQ) and Psycholoical Resilience (PR). Although conventional IQ assesses cognitive capabilities, recent research highlights the significance of emotional quotient and involvement in academic achievement. These constructs are interrelated, affecting students’ motivation, satisfaction, and overall academic engagement. In medical students, a similar study found that while most students had moderate EQ, the correlation with academic achievement was insignificant, indicating that other factors may also play a role in academic success (Winengko et al., 2024). Integrating emotional quotient training into TCM curricula could enhance students’ self-awareness and emotional regulation, potentially improving their academic engagement and performance (Latha et al., 2023). Although standard IQ is a significant indicator of cognitive aptitude, the incorporation of emotional and non-cognitive influencing factors offers a more holistic perspective on academic engagement.

*Ho1*: Emotional quotient is positively correlated with TCM students’ academic engagement.

### 2.2 The mediating role of adversity quotient

Numerous research substantiates the correlation between emotional quotient (EQ) and academic engagement among TCM students, with adversity quotient (AQ) serving as a mediator. The emotional quotient, encompassing the capacity to regulate personal emotions and comprehend those of others, is positively associated with academic engagement. The relationship is further augmented by the adversity quotient, which assesses an individual’s capacity to manage obstacles. The mediating function of adversity quotient (AQ) indicates that students possessing elevated emotional quotient (EQ) are more adept at managing obstacles, resulting in enhanced academic engagement, especially among those studying Traditional Chinese Medicine (TCM) (Cai and Guo, 2024). Incorporating emotional quotient training into curriculum can develop abilities that improve both AQ and academic engagement, resulting in superior educational outcomes (Meher et al., 2021). AQ functions as a vital intermediate linking student engagement to academic success, indicating that students with higher AQ have increased psychological resilience and participation (Cai and Guo, 2024). The correlation between emotional quotient (EQ) and adversity quotient (AQ) indicates that students with elevated emotional quotient are more adept at surmounting challenges, hence enhancing their academic engagement (Mulyasari and Maryam, 2023).

*Ho2*: The emotional quotient is positively related to adversity quotient.

*Ho3*: The adversity quotient is positively related to TCM students’ academic engagement.

*Ho4*: The emotional quotient is positively correlated with TCM students’ academic engagement with adversity quotient as the mediator.

### 2.3 The mediating role of psychological resilience

The correlation between emotional quotient (EQ) and academic engagement in Traditional Chinese Medicine (TCM) students, with psychological resilience serving as a mediator, can be analyzed within the framework of emotional quotient and its influence on academic performance. Psychological resilience is essential for improving academic engagement among students, especially those studying Traditional Chinese Medicine (TCM) (Shengyao et al., 2024). Targeted interventions to enhance emotional quotient and psychological resilience can improve learning engagement among TCM students, potentially resulting in superior academic achievement and mental health outcomes (Zeng, 2024). Enhanced emotional quotient allows students to effectively employ emotional information, leading to improved educational results, particularly in the realm of Traditional Chinese Medicine (Cai and Guo, 2024). Psychological resilience mediates the relationship between emotional quotient and academic engagement, aiding TCM major students in coping with academic stress and maintaining psychological well-being (Akyıl and İme, 2024). Psychological resilience influences learner engagement and mediates the effect of emotional quotient on academic performance (Shengyao et al., 2024).

*Ho5*: The emotional quotient is positively correlated with psychological resilience.

*Ho6*: Psychological resilience is positively related to TCM students’ academic engagement.

*Ho7*: The emotional quotient is positively correlated with TCM students’ academic engagement with psychological resilience as the mediator.

### 2.4 The moderating role of parent-student relation

The parent-student relationship can favorably regulate the association between emotional quotient (EQ) and academic engagement among TCM students. This relationship is supported by research indicating that emotional regulation and parental involvement are crucial for enhancing the academic engagement of students specializing in TCM (Sharma, 2024). The nature of parent–child relationship can profoundly impact students’ emotional regulation, therefore affecting their levels of involvement. For TCM students, cultivating a supportive parent-student relationship can enhance emotional well-being, thereby improving academic engagement and performance (Fute et al., 2024). Parental participation, encompassing emotional support and advice, positively influences students’ academic progress, which is intricately connected to engagement. Parental emotional involvement can foster a supportive learning environment and improve study habits (Mullins and Panlilio, 2023). The parent-student relationship is a vital moderator; however, factors such as parent-school communication also affect academic achievement (Mullins and Panlilio, 2023).

*Ho8*: The parent–student relation positively moderates the relation between emotional quotient and TCM students’ academic engagement.

## 3 Data collection procedure and sampling choice

The Human Research Ethics Committee at Shandong College of Traditional Chinese Medicine approved this study. Students could thereafter engage in this research based on their own volition. The informed consent statement delineated the research objectives, duration, instrument utilization, data confidentiality, and anticipated outcomes. Participants who signed this informed consent document agreed to complete the study questionnaire at the designated research location. Participants who were unable to attend in person completed the survey through an online platform. The survey was conducted from September 2024 to January 2025. Participants were administered the questionnaires in person, allowing ample time for completion and enhancing response rates. Three additional measures were implemented to enhance email survey response rates. Consequently, 53% of 800 queries (500 online and 300 offline) received responses. The online survey was distributed by email, WeChat, and QQ groups. Reminder emails were dispatched every 3 days till completion. Class supervisors urged students via class groups to submit their comments promptly. The data collection process spanned 3 months. To guarantee data accuracy and generalizability, an offline phase was executed: five classes completed the questionnaire over the course of 1 month under the oversight of class supervisors and survey administrators. For both data collection procedures, the survey administrator assessed the collected data and used SPSS to evaluate quality by excluding outliers and addressing missing data for every 200 data points. SPSS 27.0, created by IBM, was utilized to identify data collection errors and outliers. A total of 428 questionnaires were gathered.

This study employed random sampling to gather 428 completed surveys for quantitative analysis. For the qualitative part, purposive sampling was used to select experts and colleges. Random sampling entails the establishment of a target population and the probabilistic selection of samples to guarantee that each unit has an equal chance of being selected (Ahmad and Wilkins, 2024). The purposive sampling strategy applied two key selection criteria: (1) experts were required to possess a minimum of 8 years of clinical experience in psychological treatment, and (2) colleges must offer Traditional Chinese Medicine (TCM) degree programs and demonstrate documented experience in psychological education.

## 4 Instrument

**Figure 2:**
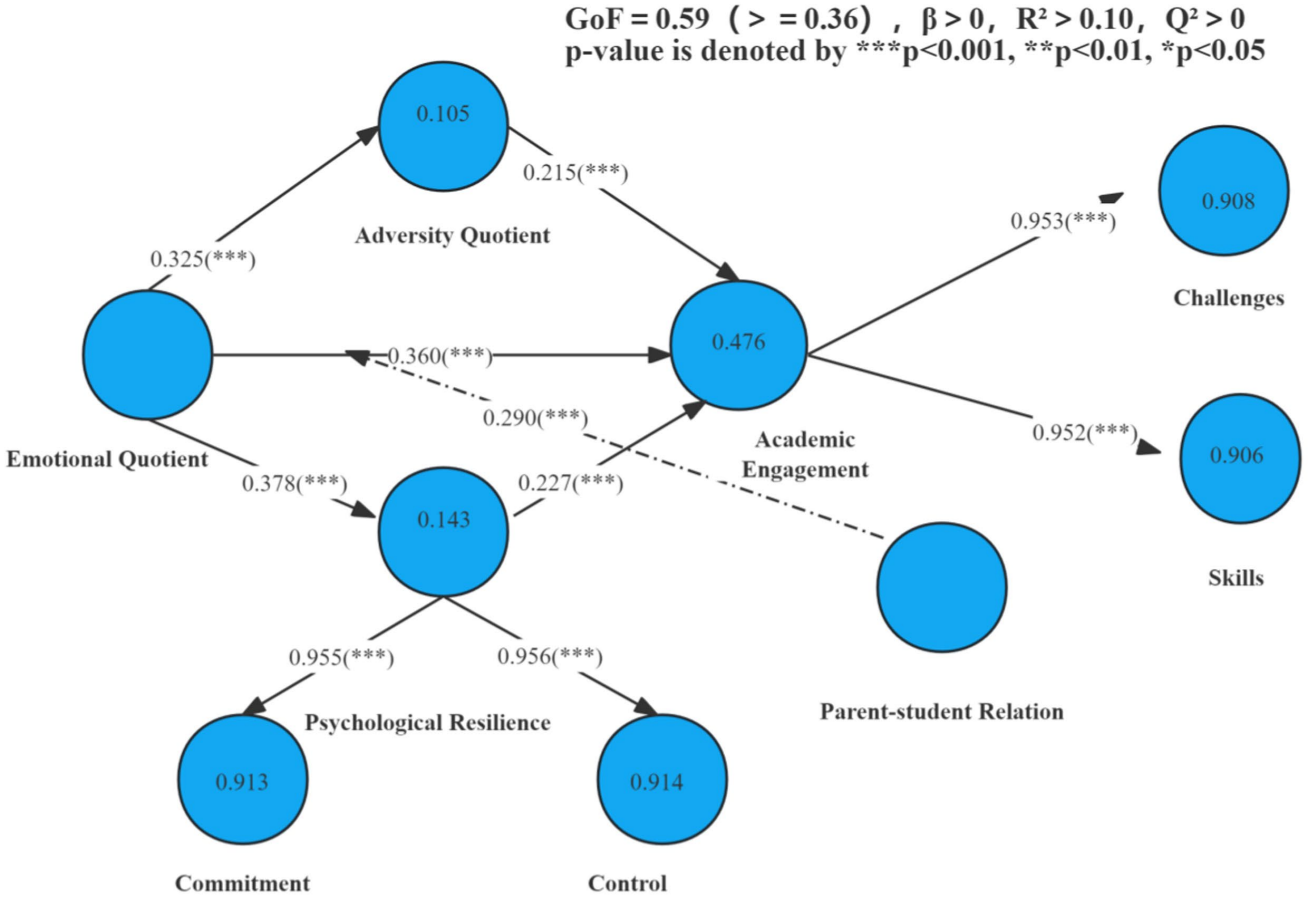
Structural model.

**Figure 3:**
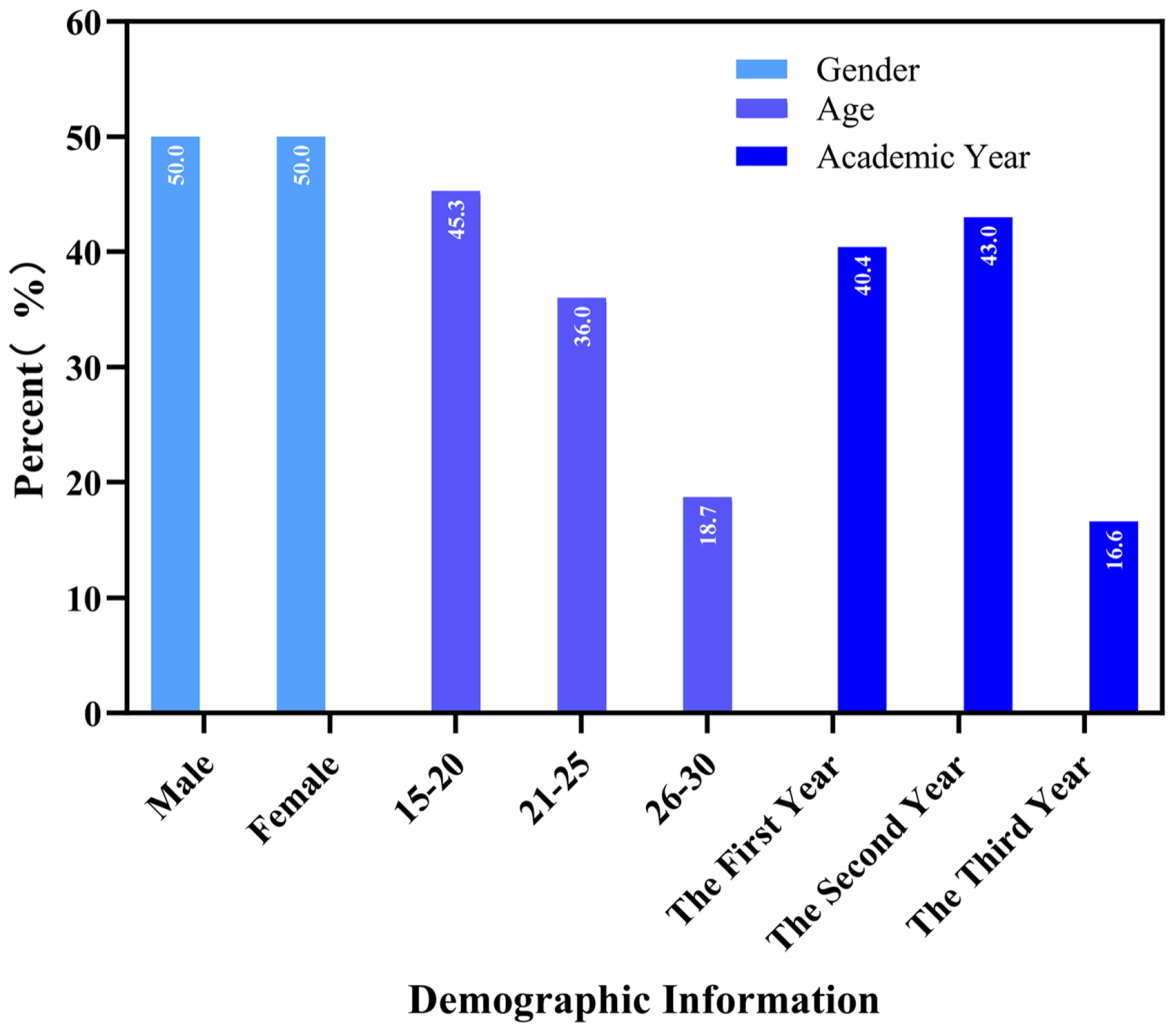
Demographic information.

The instrument applied in this study was the Academic Engagement Scale, which assessed psychosocial influencing factors and the parent-student relationship. It comprised five variables: emotional quotient (EQ), adversity quotient (AQ), psychological resilience (PR), parent-student relationship, and academic engagement, with a total of 26 items, as illustrated in Table 1. The Academic Engagement Scale integrates five dimensions adapted from existing instruments: (a) the Adversity and Emotional subscale was modified from Sudirman and Muttaqiyathun (2020)‘s Adversity Quotient Scale, with wording adjusted to fit research contexts, (b) the dimensions of psychological resilience and parent-student relationships were adapted from Self-Determination Scale (Liu and Huang, 2021), (c) the academic engagement dimension was originated from Flow Scale (Schmidt et al., 2014). After adapting the scale, we conducted content validation with two experts and five psychology researchers familiar with student engagement. The validation process involved reviewing sample items, including: ‘When facing conflicts, I remain calm and seek solutions.’ The original phrasing (‘confronting with’ and ‘composed’) was modified to ‘facing’ and ‘calm’ to enhance participant comprehension. The scale in question is both internally consistent and appropriate for the data collected, as indicated by the values of Cronbach’s Alpha (0.902) and KMO and Bartlett’s (0.918) after the validity and reliability analysis. All items demonstrated strong factor loadings (> 0.70), and HTMT values (< 0.85) confirmed discriminant validity (see Tables 2, 3) (Kim, 2023). The Goodness-of-Fit (GoF) index of 0.59 exceeded the recommended threshold of 0.36 (Figure 2) (Kim, 2023), indicating excellent model fit. Figure 3 illustrated that the participants comprised students majoring in Traditional Chinese Medicine (TCM) from various age groups (15–20: 45.3%, 21–25: 36.0%, 26–30: 18.7%) and academic years (first year: 40.4%, second year: 43.0%, third year: 16.6%). All items demonstrated strong factor loadings (> 0.70), and HTMT values (< 0.85) confirmed discriminant validity (see Tables 2, 3) (Kim, 2023).

**Table 1:** Academic engagement scale.

Variable	Dimension	Items
Emotional quotient	1. I can accurately identify my strengths and weaknesses in TCM-related learning.	
	2. In clinical practice, I can easily establish connections with patients and their families.	
	3. When making decisions, I consider both logical and emotional factors.	
	4. When facing conflicts, I remain calm and seek solutions.	
	5. When classmates share their troubles, I understand their feelings and provide support.	
Adversity quotient	1. Even when situations deteriorate, I stay calm and take action.	
	2. Even if a problem is not entirely my responsibility, I proactively participate in resolving it.	
	3. I rarely let a single failure define my overall capabilities.	
	4. Even if solving a problem requires prolonged effort, I persist with patience.	
psychological Resilience	Commitment	1. I view setbacks and challenges encountered in TCM learning as opportunities.
		2. I have strong confidence in overcoming challenges in TCM studies.
		3. I focus on completing the task at hand.
	Control	4. I believe I can shape my own destiny.
		5. I recover quickly from failures and gain valuable lessons from them.
		6. When facing critical situations, I remain calm and respond swiftly.
Parent-student relation	1. My parents encourage me to explore TCM theories beyond textbooks, which enhances my learning interest.	
	2. When I struggle with TCM concepts (e.g., Yin-Yang balance), my parents patiently help me analyze them.	
	3. My parents respect my approaches to TCM learning, even if they differ from their own views.	
	4. My parents’ trust in my TCM learning abilities boosts my confidence.	
	5. My parents focus on my personal progress in TCM studies rather than comparing me to others.	
Academic engagement	Challenges	1. The learning tasks in TCM-related courses are appropriately challenging and stimulate my thinking.
		2. Even when the TCM content is difficult, I devote effort to master it.
		3. I feel excited rather than anxious when encountering complex TCM academic problems.
	Skills	4. I can flexibly apply my knowledge to solve new academic problems.
		5. I am confident in my ability to complete TCM academic tasks efficiently.
		6. I adapt my learning strategies (e.g., reading techniques, time management) to task requirements.

**Table 2:** Convergent validity and model fit.

Items	Factors loadings	AVE	CR	Cronbach’s alpha
EQ1	0.835	0.681	0.914	0.883
EQ2	0.809			
EQ3	0.801			
EQ4	0.835			
EQ5	0.846			
AQ1	0.848	0.704	0.905	0.860
AQ2	0.835			
AQ3	0.841			
AQ4	0.833			
PR1	0.813	0.646	0.916	0.891
PR2	0.803			
PR3	0.792			
PR4	0.815			
PR5	0.810			
PR6	0.791			
COM1	0.851	0.707	0.879	0.793
COM2	0.832			
COM3	0.839			
CON1	0.824	0.709	0.880	0.795
CON2	0.850			
CON3	0.852			
PA1	0.802	0.658	0.905	0.889
PA2	0.884			
PA3	0.745			
PA4	0.743			
PA5	0.869			
AE1	0.827	0.647	0.917	0.891
AE2	0.791			
AE3	0.803			
AE4	0.789			
AE5	0.805			
AE6	0.809			
CH1	0.854	0.724	0.887	0.809
CH2	0.846			
CH3	0.852			
SK1	0.822	0.703	0.877	0.789
SK2	0.843			
SK3	0.851			
SK2	0.843			

EQ: emotional quotient; AQ: adversity quotient; PR: psychological resilience; COM: commit; CON: control; PA: parent-student relation; AE: academic engagement; CH: challenge; SK: skill.

**Table 3:** Discriminant validity.

	AE	AQ	EQ	PA	PR
AE					
AQ	0.500				
EQ	0.556	0.370			
PA	0.090	0.195	0.108		
PR	0.585	0.389	0.422	0.115	

AE: academic engagement, EQ: emotional quotient, AQ: adversity quotient; PR: psychological resilience; PA: parent-student relation.

## 5 Methodology

**Figure 4:**
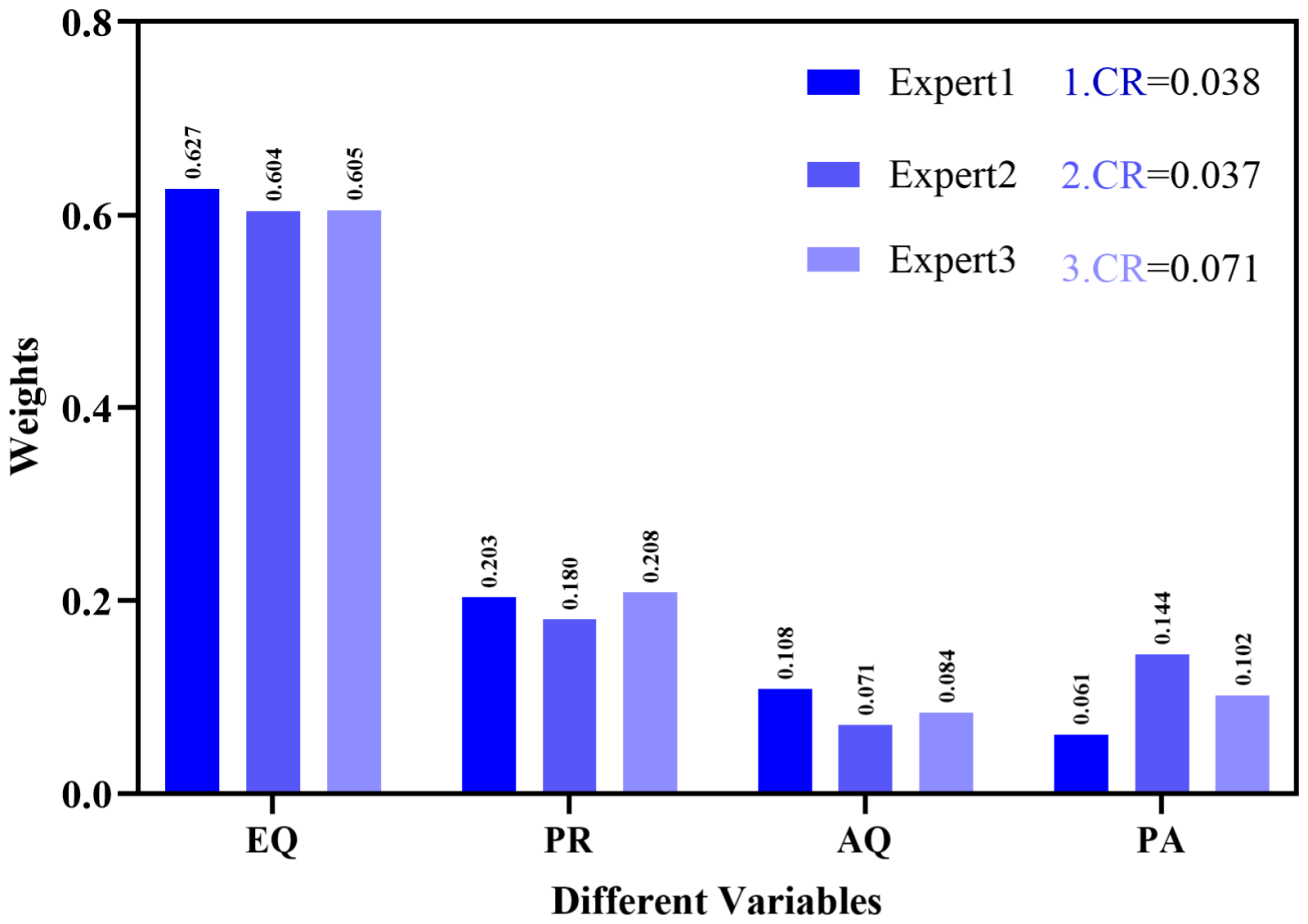
Weights determination using AHP.

**Figure 5:**
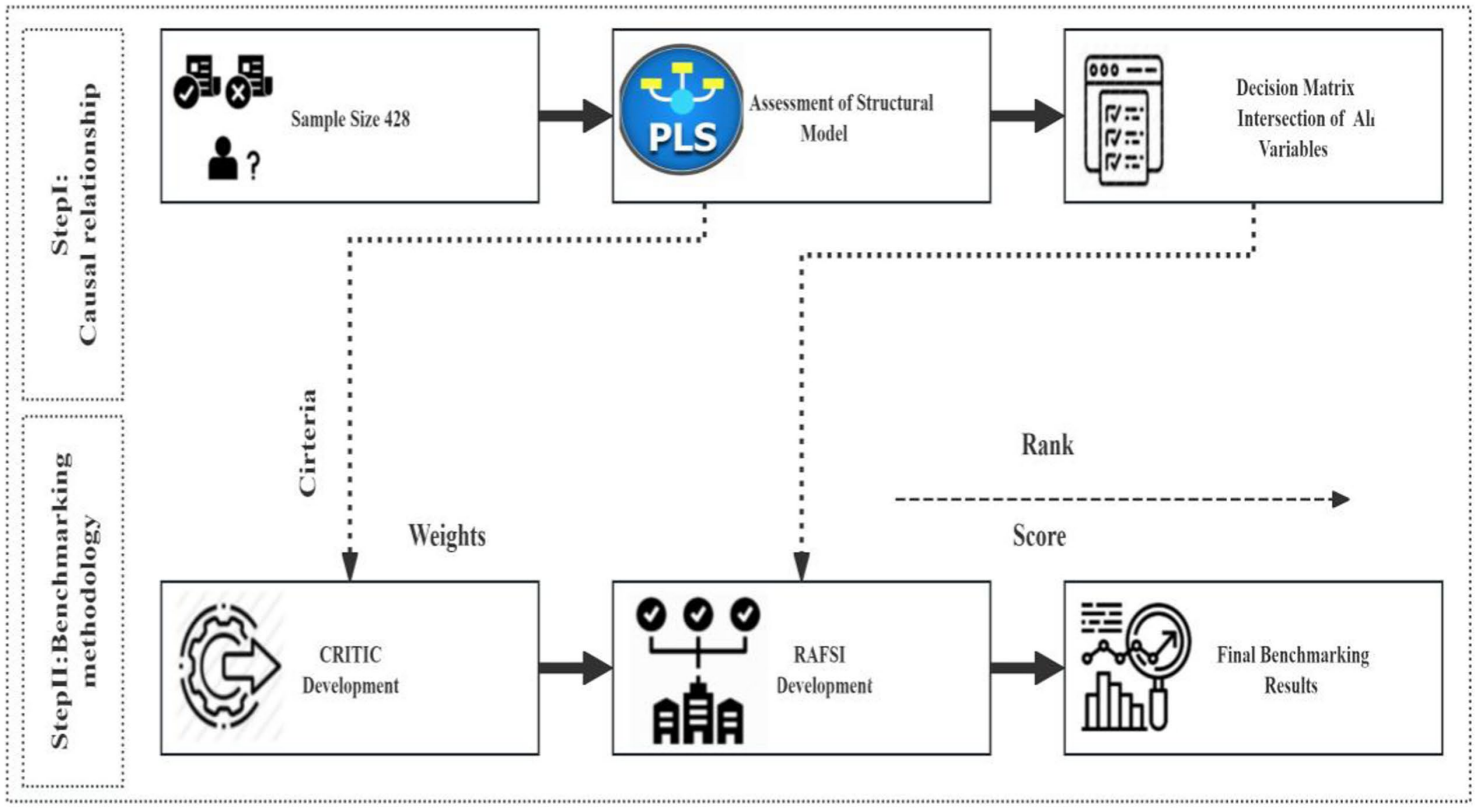
Methodology steps.

This section discussed three critical phases. The first section employed SPSS to assess the validity and reliability of the questionnaire, as well as to conduct a descriptive analysis of demographic information. The second segment employed a structural equation modeling (SEM) analytical approach. To evaluate the proposed model for this study, a structural equation modeling (SEM) method using partial least squares (PLS) was implemented, according to a two-phase analytical process. PLS-SEM is employed to model interactions among components like as beliefs, perceptions, and attitudes, which are frequently latent and assessed indirectly (Cao, 2023). The third subsection outlined the choice matrix formed from the intersection of academic engagement criteria and decision support technology. The third subsection offered a summary of the MCDM tools utilized. The Analytic Hierarchy Process (AHP) and the Vlsekriterijumska Optimizcija I Kaompromisno Resenje (VIKOR) method were utilized to assign weights to the criteria for decision support aids in academic engagement (See Figure 4). Figure 5 illustrated the methodological steps. The Analytic Hierarchy Process (AHP) is a systematic method for structuring and evaluating intricate decisions, grounded in mathematical principles and psychological theories. It assists in ascertaining the significance of each criterion in decision-making processes (Cao, 2023). VIKOR-AHP integrates the advantages of the Analytic Hierarchy Process (AHP) and the VIKOR approach, facilitating a thorough assessment of options grounded in subjective evaluations and quantifiable criteria (Taherdoost and Madanchian, 2023). This integration enables a balanced evaluation of both emotional and intellectual elements, crucial in educational environments where interpersonal interactions are key (Taherdoost and Madanchian, 2023). This flexibility guarantees that decisions are rendered with increased confidence, even in intricate situations (Cao, 2023). In contrast, although VIKOR-AHP provides a thorough methodology, many conventional MCDM techniques may be more simplistic and direct for less intricate judgments, possibly compromising depth for user-friendliness.

## 6 Data analysis

### 6.1 Structural equation model

**a) Measurement model evaluation**

Convergent validity is a crucial aspect of psychometric evaluation, indicating the degree to which two assessments of the same construct demonstrate a positive association. The validity can be assessed by various metrics, including model fit, factor loadings, Average Variance Extracted (AVE), Composite Reliability (CR), and Cronbach’s alpha (Laborde et al., 2021). An AVE of 0.50 or higher is typically considered satisfactory (Laborde et al., 2021). Composite Reliability (CR) must exceed 0.70 to affirm the construct’ s reliability (Laborde et al., 2021). As shown in Table 2, all factor loadings exceeded the acceptable threshold (≥0.70), demonstrating strong construct reliability and convergent validity. In psychometric analyses, factor loadings may further reflect semantic correlations among items, linking psychological dimensions to user-generated ratings (Stanghellini et al., 2024). A common standard for acceptable reliability is an alpha of 0.70 or above; nevertheless, multiple studies reveal values exceeding 0.80, indicating strong internal consistency (Hayat, 2024). Table 2 demonstrated convergent validity and model fit.

The Heterotrait–Monotrait ratio of correlation (HTMT) is a technique employed to evaluate discriminant validity in structural equation modeling (SEM) (see Table 3). Discriminant validity ascertains that notions intended to be disparate are genuinely distinct. The HTMT approach is seen as a more dependable alternative to conventional methods such as the Fornell–Larcker criterion and cross-loadings, which have faced criticism for their shortcomings (Dirgiatmo, 2023). The HTMT method assesses discriminant validity by comparing the HTMT value to a threshold, usually set at 0.85 or 0.90. Values beneath the cutoff signify sufficient discriminant validity (Dirgiatmo, 2023).

**b) Structural model evaluation**

To evaluate the structural model’s robustness, we employed bootstrapping to assess path significance (*p*-values) and conducted blindfolding analysis to verify predictive relevance (Q^2^). Bootstrapping includes resampling data to generate numerous simulated samples, aiding in the estimation of variability and significance of path coefficients in structural models (Lasfar and Tóth, 2024). As the Table 4 showed, the concept of blindfolding Q^2^ > 0 indicated that a predictive model possessed strong predictive power, as it suggested that the model could explain a significant portion of the variance in the outcome variable. In sectors such as healthcare, models with Q^2^ > 0 can enhance decision-making and outcomes by offering dependable forecasts (Singhal et al., 2023).

**Table 4:** Predictive relevance (*Q*^2^).

Variables	SSO	SSE	*Q*^2^ (=1-SSE/SSO)
AE	2,568	1280.049	0.502
AQ	1712	862.64	0.496
CH	1,284	728.77	0.432
CO	1,284	766.265	0.403
CON	1,284	762.14	0.406
EQ	2,140	1037.733	0.515
PA	2,140	1122.034	0.476
PR	2,568	1280.603	0.501
SK	1,284	776.077	0.396

AE: academic engagement, EQ: emotional quotient; AQ: adversity quotient; PR: psychological resilience; PA: parent-student relation; CH: challenge; CO, commit; CON, control; SK, skills.

The next stage for SEM in the PLS analysis entailed evaluating the model and verifying the hypotheses. Direct and indirect relationships will be analyzed using mediator variables, while evaluating the impact of the moderator variable on the effect of the mediator factors on the dependent variable (Maisaroh et al., 2024). The research employed bootstrapping (with 5,000 resamples and 95% confidence intervals) to estimate path coefficients, t-values, and *p*-values, thereby ensuring robust statistical inference for the structural model. Bootstrapping with an adequately large sample size (e.g., *N* = 5,000) produces findings equivalent to traditional parametric approaches, illustrating its reliability across many analytical contexts (Akter et al., 2011; Hair et al., 2017). The *β* value, standard error, T value, and *p* values shown in Table 5 and Figure 2 demonstrated that adversity quotient acted as a mediator (*p* < 0.05, *β* > 0.16, 95% CI [0.136, 0.276]) between emotional quotient and academic engagement. The psychological resilience acted as a mediator (*p* < 0.05, *β* > 0.16, 95% CI [0.202, 0.353]) between the emotional quotient and academic engagement. The emotional quotient and parent-student relationships exerted a statistically significant positive influence on students’ academic engagement (*β* = 0.227, *p* < 0.05, *t* > 1.96). The parent-student relation positively moderated the relation between emotional quotient and students’ engagement (*β* = 0.290, *p* < 0.05, *t* > 1.96). Consequently, all the hypotheses were validated. A *t*-value below 1.96 corresponds to a *p*-value beyond 0.05 in a two-tailed test, indicating non-significance (Goel et al., 2024). It is implicitly recognized that *β* > 0 is an essential requirement in these contexts (Takamizo, 2024).

**Table 5:** Assessment of structural model.

Paths	β	S.E	*T* value	*P* values
AQ - > AE	0.217	0.038	5.770	0.000
EQ - > AE	0.360	0.042	8.486	0.000
EQ - > AQ	0.325	0.050	6.431	0.000
EQ- > PR	0.379	0.048	7.844	0.000
PR - > AE	0.291	0.040	7.300	0.000
PA*EQ - > AE	0.227	0.060	3.768	0.000

AE: academic engagement; EQ: emotional quotient; AQ: adversity quotient; PR: psychological resilience; PA: parent-student relation.

The previous content primarily presented the results of the SEM analysis, whereas the next part predominantly outlined the techniques of MCDM (AHP). Initially, Structural Equation Modeling (SEM) was utilized to identify and evaluate essential variables. Thereafter, the quantitative SEM results (comprising path coefficients and factor loadings) were converted into input values for the Analytic Hierarchy Process (AHP) judgment matrix. The combined use of SEM and AHP cohesively addressed TCM students’ educational challenges. SEM identifies latent psychosocial factors (e.g., psychological resilience and parent-student relationship), while AHP prioritizes interventions (e.g., experts and colleges) using feasibility and cultural relevance (Zhang and Li, 2024). This bridges theory with practice, enabling educators to craft holistic strategies that harmonize tradition and modern academic demands, ensuring contextually adaptive solutions (Zhang and Li, 2024).

### 6.2 Multi-criteria decision making

**a) Proposed decision matrix**

The criterion was selected based on the SEM results, which provided the inputs for the MCDM, so establishing the alternatives for the 428 students and the outputs of the SEM criteria. The ELECTRE III methodology inside MCDM is utilized to rank pupils according to criteria obtained from SEM (Al-Fairouz and Abdullah, 2020). This approach addressed ambiguous and uncertain data, prevalent in student selection contexts. Consequently, this data has been utilized to rank the elements influencing students’ academic engagement. Table 5 illustrated the decision matrix.

The suggested decision-making model is based on intersecting criteria (i.e., V1, V2, V3, V4, V5, and V6) and student choices.

**b) Development**

The Analytic Hierarchy Process (AHP) and VIKOR are significant multi-criteria decision-making (MCDM) approaches that facilitate complex decision-making by evaluating options based on several criteria. The integrated AHP-VIKOR methodology capitalizes on the advantages of both techniques: AHP formulates hierarchical frameworks and ascertains criterion weights via expert evaluations, whereas VIKOR systematically ranks intervention alternatives by assessing their proximity to optimal results across conflicting priorities. AHP systematically weights noncognitive aspects, while VIKOR offers thorough intervention assessments, providing practical and theoretically robust solutions vital for formulating targeted academic and mental health support programs. The Analytic Hierarchy Process (AHP) is predominantly utilized for determining the weights of criteria via pairwise comparisons (Atlı, 2024) and is widely applied in several domains such as machine selection, supplier selection, and resource prioritization in healthcare. It provides a structured framework for decision-making by quantifying and evaluating elements related to overarching objectives and potential solutions (Srebrenkoska et al., 2023).

**c) Analytic hierarchy process weighting method for weighting criteria**

This section outlined the steps of the AHP approach for evaluating criteria as follows.

Step 1: The hierarchy includes the Decision Matrix (DM), and the conditions specified inside each DM to determine the relationship between the criteria in the students’ DM and the factors affecting their academic engagement, with prioritizing performed to subjectively assign weights.

Step 2: The AHP constructs a pairwise comparison matrix using Equation 1 to ascertain a weighting decision.

(1) $$A=\left(\begin{array}{ccc} X_{11} & \cdots & X_{1n}\\ \vdots & \ddots & \vdots \\ X_{n1} & \cdots & X_{\mathrm{nn}} \end{array}\right)$$

Where Xii = 1, Xij = $\frac{1}{X_{\mathrm{ij}.}}$

Step 3: This step delineates the development of the peer-review questionnaire according to the criteria for each judgment matrix associated with the prioritization of students’ academic engagement and the experts.

Step 4: At this juncture, each component in matrix A (1) is standardized to generate the normalized matrix Anorm, denoted as Anorm (aij), in the following manner:

(2) $$\mathrm{aij}=\frac{X_{\mathrm{ij}}}{\sum_{i=1}^{n}X_{\mathrm{ij}}}$$

$$\mathrm{Anorm}=\left(\begin{array}{ccc} a_{11} & \cdots & a_{\mathrm{in}}\\ \vdots & \ddots & \vdots \\ a_{n1} & \cdots & a_{\mathrm{nn}} \end{array}\right)$$

A (xij) is defined by Equation 2.

Step 5: This phase involves AHP pairwise analysis employing Equation 3, interpreting decisions, and assigning weights to each priority decision-maker.

(3) $$\mathrm{Wi}=\frac{\sum_{j=1}^{n}a_{\mathrm{ij}}}{n}\;\mathrm{and}\;\overset{n}{\underset{j=1}{\sum }}W_{i}=1$$

Step 6: This step including Equations 4–6 entails evaluating the composite reliability (CR) of the pairwise comparison matrix as outlined below.

(4) $$\mathrm{CR}=\frac{\mathrm{CI}}{\mathrm{RI}}$$

(5) $$\mathrm{CI}=\frac{⋋\max-n}{n-1}$$

(6) $$\mathrm{RI}=\frac{1.98\left(n-1\right)}{n}\mathrm{CI}$$

A pairwise comparison matrix is deemed admissible if its consistency ratio (CR) is 10% or 0.1 or lower; otherwise, it will be rejected.

**d) Weights determination using analytic hierarchy process**

This section specifies the criteria weights of the decision maker (emotional quotient, adversity quotient, psychological resilience, and parent-student relations), utilized to prioritize students’ academic engagement through the AHP process.

Three educational experts were asked to assess different components through comparative analyses. Due to the consistency problem, they were requested two additional times. The weight results were displayed in Figure 4.

The weights of the academic engagement criterion for students, assessed by three experts, were illustrated in Figure 4. The primary expert ascribed the greatest significance to emotional quotient, the secondary expert to adversity quotient, and the tertiary expert to psychological resilience; conversely, the parent-student relationship received the least importance. The weights supplied by experts were within acceptable parameters, as demonstrated by consistency findings below 0.1.

**e) Vlsekriterijumska Optimizcija I Kaompromisno Resenje method for prioritizing students’ academic engagement**

The VIKOR method is utilized to rank the academic engagement of students majoring in TCM. The VIKOR methodology comprises specific phases (Gul, 2024).

Step 1: Identify the optimal f* and suboptimal f- values for each criterion within DM, where i = 1, 2, n. If the function represents:

$$\;f_{i}^{*}=\max_{j}\;f_{\mathrm{ij}},f_{i}^{-}=\min_{j}f_{\mathrm{ij}}$$

where i = 1, 2,., n.

Step 2: The Analytic Hierarchy Process (AHP) is employed to calculate the evaluation criteria for each priority decision maker (DM). A set of weights w = {w1, w2, w3, wj, wn} from the experts is integrated into the decision-making process, with the total summing to 1. The resultant matrix can alternatively be determined as seen in the following Equation 7:

(7) $$\mathrm{WM}=w_{i}\frac{f_{i}^{*}-f_{\mathrm{ij}}}{f_{i}^{*}-f_{i}^{-}}$$

$$\begin{array}{ccc} \frac{w_{1}\left(f_{1}^{*}-f_{11}\right)}{f_{i}^{*}-f_{i}^{-}} & \cdots & \frac{w_{i}\left(f_{i}^{*}-f_{ij}\right)}{f_{i}^{*}-f_{i}^{-}}\\ \vdots & \ddots & \vdots \\ \frac{w_{1}\left(f_{i}^{*}-f_{31}\right)}{f_{i}^{*}-f_{i}^{-}} & \cdots & \frac{w_{i}\left(f_{i}^{*}-f_{ij}\right)}{f_{i}^{*}-f_{i}^{-}} \end{array}$$

Step 3: In this step, the Sj and Rj values, with j = 1,2,3,., J and i = 1,2,3,., n, can be calculated using the following Equations 8 and 9:

(8) $$\mathrm{Sj}=\sum_{i=1}^{n}w_{i}\frac{f_{i}^{*}-f_{\mathrm{ij}}}{f_{i}^{*}-f_{i}^{-}}$$

(9) $$\mathrm{Rj}=\max_{i}w_{i}\frac{f_{i}^{*}-f_{\mathrm{ij}}}{f_{i}^{*}-f_{i}^{-}}$$

Step 4: Determine the values of Qj, where j = (1,2,. J), using the following Equation 10:

(10) $$Q_{j}=\frac{V\left(S_{j}-S^{*}\right)}{S^{-}-S^{*}}+\frac{\left(1-V\right)\left(R_{j}-R^{*}\right)}{R^{-}-R^{*}}$$

Where

$$\begin{array}{cc} S^{*} & =\min_{j}S_{j},S^{-}=\max_{j}S_{j}\\ R^{*} & =\min_{j}R_{j},R^{-}=\max_{j}R_{j} \end{array}$$

v is defined as the weight of the ‘most criteria’s strategy (or ‘highest group utility’); in this instance, *v* = 0.5. The concept of a decision-maker exhibiting neutrality, as indicated by a value of *v* = 0.5, suggests a balanced approach to decision-making, where no clear preference for one alternative over another is established. This neutrality can enhance the objectivity of the findings and the transparency of the methodology employed in the study (Gul, 2024). The adoption of *v* = 0.5 in AHP-VIKOR applications ensures equilibrium between collective priorities and individual needs. In psychological resilience, this balances adaptive capacity (group utility) and trauma recovery (individual regret) (Iacoviello and Charney, 2023). This study employed *v* = 0.5 as the compromise coefficient in the VIKOR method to maintain an objective balance between maximizing group utility and minimizing individual regret.

Step 5: The alternative focus, particularly on students’ academic engagement, may now be emphasized. The operation is performed by organizing the Q-values in an ascending sequence.

**f) The ranking by VIKOR methodology analysis**

The weights derived from the AHP analysis conducted by three experts can be applied to each criterion within the internal group utilizing the VIKOR approach. The aim of VIKOR is to provide solutions that are satisfactory to all stakeholders, while considering the preferences of both individuals and the collective (Gul, 2024). The questionnaires were sent throughout five separate colleges, and the average of several parameters was calculated. Table 6 illustrated the college rankings alongside the differing Q values. The inference that college 5 occupied the top position and college 3 the second position could be derived from Table 6. College 5 demonstrated significantly stronger cultivation of students’ psychological well-being compared to the other institutions. Conversely, College 2 allocated minimal attention to fostering emotional quotient cultivation. The VIKOR rankings revealed practical insights: College 5 excelled in psychological support (model for replication), College 3 showed moderate effectiveness, while College 2 lagged in emotional development. These results guided targeted improvements—reallocating resources to weak areas, adopting top performers’ strategies, and enhancing faculty training. The alignment of AHP-derived weights with outcomes validated the method and offered actionable steps to optimize TCM education.

**g) Sensitivity analysis**

**Table 6:** Individual VIKOR prioritization for five colleges’ academic engagement.

Expert 1			Expert 2			Expert 3		
ID	Q	Order	ID	Q	Order	ID	Q	Order
1	0.149	3	1	0.146	4	1	0.130	3
2	1.000	5	2	1.000	5	2	1.000	5
3	0.031	2	3	0.002	1	3	0.044	2
4	0.228	4	4	0.114	3	4	0.177	4
5	0.007	1	5	0.025	2	5	0.000	1

The ultimate average expert weight was first determined by calculating the average weights of three experts. The sensitivity analysis was calculated by the average experts. Sensitivity analysis, specifically, aids in comprehending the robustness of decision findings when expert weights are modified (Yagmahan and Yılmaz, 2022).

Step 1: Identify the $\bar{\omega }$ values for the average experts weights, where i = 1, 2, n. The related function was listed as below Equation 11:

(11) $$\bar{\omega }=\frac{1}{3}\overset{3}{\underset{i=1}{\sum }}\omega_{i}$$

Step 2: The weights of other criteria also change when a criterion’s weight shifts from Xi to Yi. The weight redistribution among other criteria resulting from changing one criterion’s weight from Xᵢ to Yᵢ followed this Equation 12:

(12) Yj=(1−Yi)Xj/(1−Xi)(i,j=1,2……n,i≠j)

Step 3: In the formula, X and Y denote the original weight vector and the modified weight vector of the criterion, respectively, with the updated vector Y remaining normalized, as the below Equation 13 demonstrated:

(13) $$\overset{m}{\underset{j=1}{\sum }}Y_{j}=Y_{i}+\overset{m}{\underset{\begin{array}{c} j=1\\ j\neq i \end{array}}{\sum }}\left(1-Y_{i}\right)\frac{X_{j}}{1-X_{i}}=Y_{i}+\frac{1-Y_{i}}{1-X_{i}}\times \left(1-X_{j}\right)=1$$

**Figure 6:**
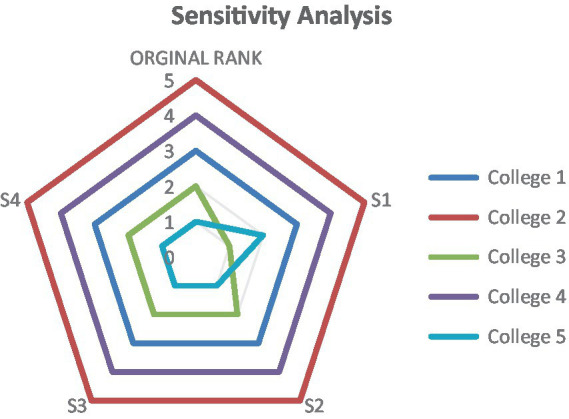
Sensitivity analysis.

In this study, we systematically adjusted the average weights of three expert-defined criteria by ±10% through simultaneous multi-criteria perturbation rather than single-parameter variation, and monitored the ranking stability across the five participating colleges under these modified weighting schemes. Researchers have preferred sensitivity analysis to improve the model’s accuracy and stability in applications. If the outcomes are consistent across parameter adjustments, the model can be considered robust (Yagmahan and Yılmaz, 2022). Following the calculation procedures outlined above, the rankings based on the Q values are presented in Figure 6. To test the robustness of the model, each original criterion weight was individually increased by 10%, while the weights of the remaining three variables were adjusted proportionally to maintain normalization.

**Figure 7:**
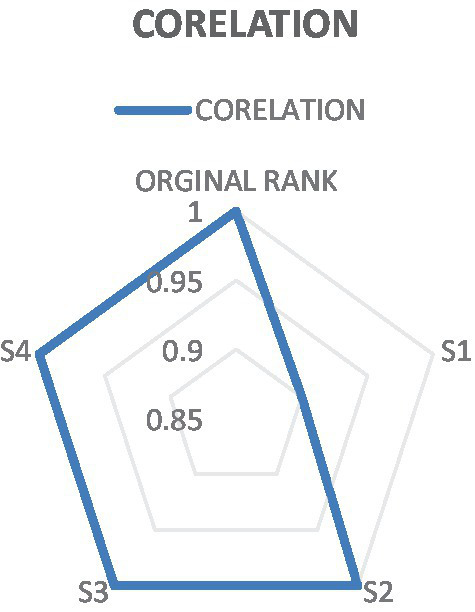
Spearman correlation coefficient.

As shown in Figure 6, Colleges 1, 2, 3, 4, and 5 maintained consistent rankings across Scenarios 2, 3, and 4. However, College 3 was the only exception in Scenario 1, where it ranked first—unlike the other three colleges. Among the remaining colleges, College 5 held the top position in the other scenarios. The results demonstrated that the final rankings almost remained unchanged, as illustrated in Figure 6, confirming the stability and robustness of the research findings. Ultimately, Spearman’s rank served as the conclusive validation methodology to assess our outcomes. Furthermore, it was acknowledged as a robust method for assessing correlations among a set of variables (Muhsen et al., 2024). Figure 7 displayed the findings of the correlation analysis for the ranks of Colleges. The investigation demonstrated significant correlation coefficients, with the minimum value recorded at 90% in a single instance, while all other values reached 100%.

## 7 Discussion

The research objectives and the corresponding procedures employed to attain them were outlined in Table 7. The primary purpose is to identify all elements influencing students’ academic engagement using the SmartPLS analytic method, with all eight hypotheses being tested. The second purpose is to emphasize the importance of noncognitive influencing elements through the application of the AHP analysis approach. This analysis indicates that the emotional quotient is the primary influential component, succeeded by psychological resilience, adversity quotient, and parent-student relationship. The results showed that the lower influence of parent-student relationships reflected: (1) expert prioritization of intrinsic student factors (EQ and psychological resilience) over external supports, and (2) cultural perceptions in TCM education valuing self-cultivation. The third purpose is to examine the practical application of psychological education in five colleges with the application of VIKOR analysis method. The conclusion indicated that College 5 prioritized psychological education far more than College 2, which afforded the least attention to this aspect of students’ education.

**Table 7:** Research objectives and findings summary.

Objectives	Method	Results
To explore the noncognitive influencing factors related to the enhancement of students’ academic engagement.	SmartPLS analysis	All contributing aspects, including emotional quotient, adversity quotient, psychological resilience, and the parent-student relationship, related to the development of students’ academic engagement were identified.
To elucidate and prioritize the significance of noncognitive influencing elements.	Analytic Hierarchy Process Analysis Method	The emotional quotient is the primary influencing factor, followed by psychological resilience, adversity quotient, and the parent-student interaction.
To examine whether colleges effectively emphasize and implement psychological education.	Vlsekriterijumska Optimizcija I Kaompromisno Resenje Method	College 5 enhances students’ psychological well-being more effectively than the preceding four institutions. Conversely, College 2 afforded diminished emphasis on emotional development.

The theoretical framework, validated through structural equation modeling of 428 questionnaires, revealed that emotional quotient positively correlates with academic engagement, influenced by psychological resilience and adversity quotient, while parent-student relationships moderate this link. Flow theory further explains this dynamic, as deep task absorption enhances engagement and noncognitive factors such as psychological resilience and adversity quotient in TCM students, enriching their learning experiences (Dominek, 2023). The AHP analysis methodology was employed in the second phase to assess the judgments of three experts and ascertain the weights of various factors. The Q value was derived from five institutions, and the ranking of four factors was established using the VIKOR analysis approach. The research findings aligned with prior studies. Students possessing heightened emotional quotient are more likely to participate, so increasing their confidence in academic capabilities and subsequently improving performance (Bonilla-Yucailla et al., 2022). Favorable emotions, a component of emotional quotient, significantly predict learning engagement, highlighting the need of fostering a favorable emotional climate in educational settings (Zhoc et al., 2021). The primary focus of prior study was the linear relationship among emotional quotient, adversity quotient, psychological resilience, parent-student relations, and academic engagement. This study further investigated the nonlinear correlation among several factors. Furthermore, it incorporated evaluations from students, experts, and colleges to objectively analyze the linkages from both theoretical and practical viewpoints.

### 7.1 Theoretical implications

The relationship between emotional quotient (EQ) and college students’ academic engagement is intricate, highlighting the importance of psychological resilience in both academic and personal domains. Research indicates that a heightened emotional quotient (EQ) might enhance student engagement and academic achievement, both of which are critical components of academic engagement (Thomas and Allen, 2020). Enhanced psychological resilience allows students to effectively use social support, leading to improved emotional well-being and academic achievement (Salim and Sarajar, 2024). This study sought to examine the impact of psychological resilience and social support on the subjective academic engagement of college students (Salim and Sarajar, 2024). The relationship between emotional quotient (EQ) and academic engagement can be significantly influenced by the parent-student relationship, which acts as a moderating variable (Puiu et al., 2024). This study investigated the linear and nonlinear relationships between emotional quotient and students’ academic engagement, with adversity quotient and psychological resilience serving as mediators, and parent-student relations acting as a moderator, in contrast to extensive research examining the direct connections among emotional quotient, adversity quotient, psychological resilience, parent-student relations, and academic engagement (Puiu et al., 2024). This study established a new theoretical model. This research utilized the Analytic Hierarchy Process (AHP) and the Vlsekriterijumska Optimizcija I Kaompromisno Resenje (VIKOR) method to analyze non-linear correlations among several variables related to students’ academic engagement. A sensitivity analysis was conducted to verify the robustness of the results, confirming that the outcomes remained stable despite minor variations in expert-assigned weights. It integrated cognitive and non-cognitive elements affecting the improvement of students’ academic engagement and addressed the enduring topic of why individuals with high intellect often underperformed academically.

### 7.2 Practical implications and limitations

This study included two variables—adversity quotient and psychological resilience—that enhanced the current research on the influence of emotional quotient on academic engagement. Research reveals adversity quotient (AQ) as a key mediator between student engagement and academic success, with higher AQ predicting better outcomes (Cai and Guo, 2024). Parent-student relationships further moderate the EQ-engagement link, underscoring the value of targeted support for sensitive students. These findings advocate for comprehensive interventions combining AQ development (e.g., psychological resilience training) and family-inclusive strategies to foster engagement. By prioritizing adversity quotient (AQ), EQ, and relational support, educators can create nurturing environments that enhance academic performance while equipping students to navigate challenges effectively. Tailoring these strategies ensures all students—particularly in TCM—can experience flow, fostering motivation, deep engagement, and long-term success (Dominek, 2023).

Secondly, incorporate EQ or AQ modules that link Yin-Yang philosophy to emotional management. Utilize role-playing, introspective journaling, and Qi Gong to cultivate empathy, self-awareness, and psychological resilience. Encourage collaborative learning through feedback from peers and parents. Educate instructors in emotional coaching to promptly assess students’ subtle emotional fluctuations. Incorporate clinical case studies on coping with adversity to enhance students’ adversity quotient. These modifications integrate TCM’s comprehensive methodology with emotional growth, promoting involvement, autonomy, and resilience via structured social and academic assistance. The enhancement of students’ positive emotions will lead to improved engagement, fostering holistic psychological growth and, in turn, higher positive feelings, so supporting an elevated sense of autonomy in their self-development and self-realization.

This study has many limitations. The cross-sectional approach limits causal assertions between emotional quotient (EQ) and academic engagement; longitudinal data are necessary to prove temporal correlations. Secondly, self-report bias may exaggerate observed correlations, as participants’ replies could be influenced by social desirability or recall biases instead of objective behaviors. The sample consists solely of TCM students, limiting cultural and disciplinary generalization. Although the findings provide insights into Traditional Chinese Medicine major, they may not be applicable to varied educational or cultural environments. Moreover, unmeasured variables (e.g., pedagogical approaches and social contexts) may affect research outcomes. Future research ought to employ multidisciplinary, longitudinal methodologies with varied samples, integrate objective measurements (e.g., behavioral evaluations), and investigate cultural heterogeneity to enhance validity and applicability.

## 8 Conclusion

This study examined linear/non-linear relationships between emotional quotient (EQ) and academic engagement, incorporating psychological resilience (PR) as a mediator and parent-student relationships as moderator. Applying flow theory, we identified optimal EQ thresholds where students experience heightened engagement. Findings reveal targeted intervention points for TCM students, suggesting balanced EQ development can foster flow states, thereby mitigating stress and negative emotions through improved self-regulation and supportive relationships.

For educators, integrating evidence-based resilience-building practices (e.g., mindfulness training) into curricula can enhance emotional adaptability. Policymakers should prioritize institutional reforms to cultivate supportive learning environments—reducing stressors while promoting intellectual growth. Parents play a critical moderating role; their emotional engagement bolsters students’ adversity quotient (AQ) and mitigates academic pressure. By addressing these interconnected factors, TCM programs can optimize both psychological well-being and academic performance, ensuring students thrive amid rigorous training demands.

## Data Availability

The datasets presented in this study can be found in online repositories. The names of the repository/repositories and accession number(s) can be found in the article/supplementary material.
